# Combination Biomarker of Immune Checkpoints Predict Prognosis of Urothelial Carcinoma

**DOI:** 10.3390/biomedicines10010008

**Published:** 2021-12-22

**Authors:** Chung-Ying Tsai, Hsiang-Cheng Chi, Ren-Chin Wu, Cheng-Hao Weng, Tzong-Shyuan Tai, Chan-Yu Lin, Tai-Di Chen, Ya-Hui Wang, Li-Fang Chou, Shen-Hsing Hsu, Po-Hung Lin, See-Tong Pang, Hung-Yu Yang

**Affiliations:** 1 Kidney Research Center, Department of Nephrology, Chang Gung Memorial Hospital, College of Medicine, Chang Gung University, Taoyuan 333, Taiwan; cytsai0616@gmail.com (C.-Y.T.); drweng@seed.net.tw (C.-H.W.); r5234@adm.cgmh.org.tw (C.-Y.L.); d928209@gmail.com (L.-F.C.); d938208@gmail.com (S.-H.H.); 2 Graduate Institute of Integrated Medicine, China Medical University, Taichung 404, Taiwan; hcchi@cmu.edu.tw; 3 Chinese Medicine Research Center, China Medical University, Taichung 404, Taiwan; 4 Department of Pathology, Chang Gung Memorial Hospital, Taoyuan 333, Taiwan; renchin.wu@gmail.com (R.-C.W.); 8902028@cgmh.org.tw (T.-D.C.); 5 Department of Pathology, Johns Hopkins Medical Institutions, Baltimore, MD 21205, USA; 6 Advanced Immunology Laboratory, Chang Gung Memorial Hospital, Taoyuan 333, Taiwan; imb503@gmail.com; 7 Institute of Stem Cell and Translational Cancer Research, Chang Gung Memorial Hospital, Chang Gung University, Taoyuan 333, Taiwan; yhwang@cgmh.org.tw; 8 Graduate Institute of Clinical Medical Science, College of Medicine, Chang Gung University, Taoyuan 333, Taiwan; m7587@cgmh.org.tw (P.-H.L.); pst64lab@gmail.com (S.-T.P.); 9 School of Medicine, College of Medicine, Chang Gung University, Taoyuan 333, Taiwan; 10 Division of Urology, Department of Surgery, Chang Gung Memorial Hospital, Taoyuan 333, Taiwan; 11 Department of Health Policy and Management, Johns Hopkins Bloomberg School of Public Health, Baltimore, MD 21205, USA

**Keywords:** urothelial carcinoma, regulatory T cells, immune checkpoints, *CD276*, *TIM-3*

## Abstract

In contrast to Western counties, the incidence of urothelial carcinoma (UC) remains mar-edly elevated in Taiwan. Regulatory T cells (Tregs) play a crucial role in limiting immune responses within the tumor microenvironment. To elucidate the relationship between immune checkpoints in the tumor immune microenvironment and UC progression, we utilize the Gene Expression Omnibus (GEO) to analyze a microarray obtained from 308 patients with UC. We observed that the expression level of *CD276* or *TIM-3* was positively correlated with late-stage UC and poor prognosis. Patients with simultaneously high *CD276* and *TIM-3* expression in tumors have significantly reduced both univariate and multivariate survival, indicating that mRNA levels of these immune checkpoints could be independent prognostic biomarkers for UC overall survival and recurrence. Our cohort study showed rare CD8+ cytotoxic T-cells and Tregs infiltration during early-stage UC-known as cold tumors. Approximately 30% of late-stage tumors exhibited highly infiltrated cytotoxic T cells with high PD-1 and FOXP3 expression, which implied that cytotoxic T cells were inhibited in the advanced UC microenvironment. Collectively, our findings provide a better prognosis prediction by combined immune checkpoint biomarkers and a basis for early-stage UC standard treatment to convert cold tumors into hot tumors, followed by immune checkpoint therapy.

## 1. Introduction

Managing UC remains a major challenge that needs to be urgently addressed. Tumors are unique and complex immune microenvironments, exhibiting lymphocytes that infiltrate into neoplastic lesions, called tumor-infiltrating lymphocytes (TILs). TILs play an active role in facilitating or inhibiting tumor growth and invasion [1]. In addition, CD8 (killer) T cells are responsible for substantial antitumor effects, along with other participating immune cells.

Regulatory T cells (Tregs) play a key role as regulators of the immune microenvironment by inhibiting aberrant immune responses [2]. Tregs typically express the forkhead family transcription factor *Foxp3*, which is indispensable for Treg development and function. A paucity of Treg function and/or number has been reported under autoimmune states [2,3]. Conversely, Tregs appear to play a major role in the inability to immunize against evolving tumors [4]. A strong correlation between Treg frequency and clinical outcome has been reported in patients with ovarian, lung, pancreatic, and breast cancer [5]. A recent report has strongly suggested that Tregs may be crucial in the progression of UC [6].

Tregs can suppress effector T cells via several mechanisms. First, Treg cells can kill T effector cells directly in culture by granzyme B and perforin [7,8]. Additionally, Tregs can also suppress T effectors by delivering large amounts of cAMP through contact [9] or by suppressing cytokines, such as IL-10 [10], IL-9, IL-35 [11], and transforming growth factor (TGF)-β [12]. Lastly, surface molecules on Tregs afford another suppressive mechanism that modulates dendritic cells (DCs), as well as effector T or B cells. CD 39 and CD73 catalyze the generation of adenosine, which suppresses effector T cells by binding to A2A receptors [13]. The B7 family, including *CD274*, *CD273*, *ICOSL*, *CD276 (B7-H3)*, and *CD80*, also plays a pivotal role in regulating T-cell immune responses. *CD276* is highly overexpressed on a wide range of human malignant tumor tissues and correlated with poor prognosis, including pancreatic cancer, breast cancer, colon cancer, lung cancer…etc. [14]. *CD276* is also associated with a lower number of tumor-infiltrating lymphocytes, implying a role for CD276 in tumor immune evasion and inhibition of T cell antitumor immunit [15] y. To date, the role of *CD276* on the survival and relapse of UC patients is still unknown.

Cytotoxic T-lymphocyte antigen 4 (CTLA4) on Tregs can stimulate antigen-presenting cells to increase the activity of indoleamine 2, 3-dioxygenase (IDO), a potent immunosuppressive enzyme [16]. A recent study has revealed that Tregs use programmed death-ligand 1 (PD-L1) to suppress autoreactive B cells [17]. Inducible T-cell costimulatory receptor (ICOS or CD278) is another Treg surface molecule belonging to the CD28 protein family. A novel Ig family member, TIGIT, which is highly expressed on Tregs, reportedly induces DCs to produce IL-10 and TGF-β [18]. TIM3 (T-cell immunoglobulin domain and mucin domain 3, also known as *HAVCR2*) and its ligand galectin-9 have inhibitory roles of effector T cells response. Several studies have indicated that TIM3^+^ Treg cells exhibit greater suppression function than TIM3^−^ Treg cells [19,20].

In 2013, cancer immunotherapy was named “Breakthrough of the Year” by *Science* [21] partly due to the clinical success of blocking antibodies against programmed cell death protein 1 (PD-1) and PD-L1 in different cancers [22]. Inhibition of critical immune checkpoints (ICIs) leads to enhanced anti-tumor immunity and suppressed tumor growth by enhancing CD8+ tumor infiltrating T cells (TILs) and decreasing regulatory T cells (Tregs) in the tumor microenvironment. However, the therapeutic efficacy of ICIs is dependent on the number of TILs in tumor. Patients expressing low levels of TILs is defined as cold tumor, typically less responsive to immunotherapy treatment using ICIs [23]. The relationship between UC stage and immune checkpoint profiling as well as TILs infiltration remains unclear. In the present study, we focused on immune checkpoint profiling and combined biomarkers to predict UC patient outcome. We also want to explore the immune profiling in UC with different stage to provide a rationale of treatment for UC with different stage.

## 2. Materials and Methods

### 2.1. Subjects

Formalin-fixed, paraffin-embedded (FFPE) specimens from 80 UC patients diagnosed pathologically with at Chang Gung Memorial Hospital (CGMH) from 2012 to 2017 were obtained from tissue bank of CGMH. The study protocol was approved by the Medical Ethics and Human Clinical Trial Committee of the CGMH (IRB NO 20150160B0, accessed on 14 May 2015).

We analyzed the transcriptome database from the Gene Expression Omnibus (GEO) dataset (GSE32894, GSE13507 and GSE31684) [24,25,26,27] to explore the clinical significance of immune checkpoints. Furthermore, we analyzed overall survival using Kaplan–Meier survival analysis. Finally, the correlation coefficient was tested using the Pearson correlation analysis.

### 2.2. Immunohistochemistry (IHC)

The following commercial primary antibodies were used: Anti-CD8 (ab4055, ABCAM, Cambridge, MA, USA), Anti-FOXP3 (ab20034, ABCAM, Cambridge, MA, USA), Anti-PD-1 (ab52587, ABCAM, Cambridge, MA, USA). The avidin-biotin complex method and scoring formula were performed as previously described [28]. T-cell types in each compartment were quantitated numerically by blinded analysis of 5 hpf (high power fields) (0.0028 mm ^2^/hpf) and displayed as the average number of stained cells per hpf.

### 2.3. Statistical Analysis

The Mann–Whitney U-test or Fisher’s exact test was used for between-group comparisons, where appropriate, and the correlation between the results, obtained with the two different analyses, was analyzed using Spearman’s test. Follow-up studies were performed until the time of writing this report or patient death. The cancer-specific survival outcome was evaluated using the Kaplan–Meier method for all patients, except those who died from surgical complications. The log-rank test was used to compare the prognostic significance of individual variables on survival. Multivariate analysis was performed using the Cox proportional hazards model to identify independent predictors of survival. All graphing and statistical analyses were performed using either GraphPad Prism software version 9 (GraphPad Software, Inc., San Diego, CA, USA) or SPSS version 20 statistical software (SPSS Inc., Chicago, IL, USA). Statistical significance was set at *p* < 0.05.

## 3. Results

**Figure 1:**
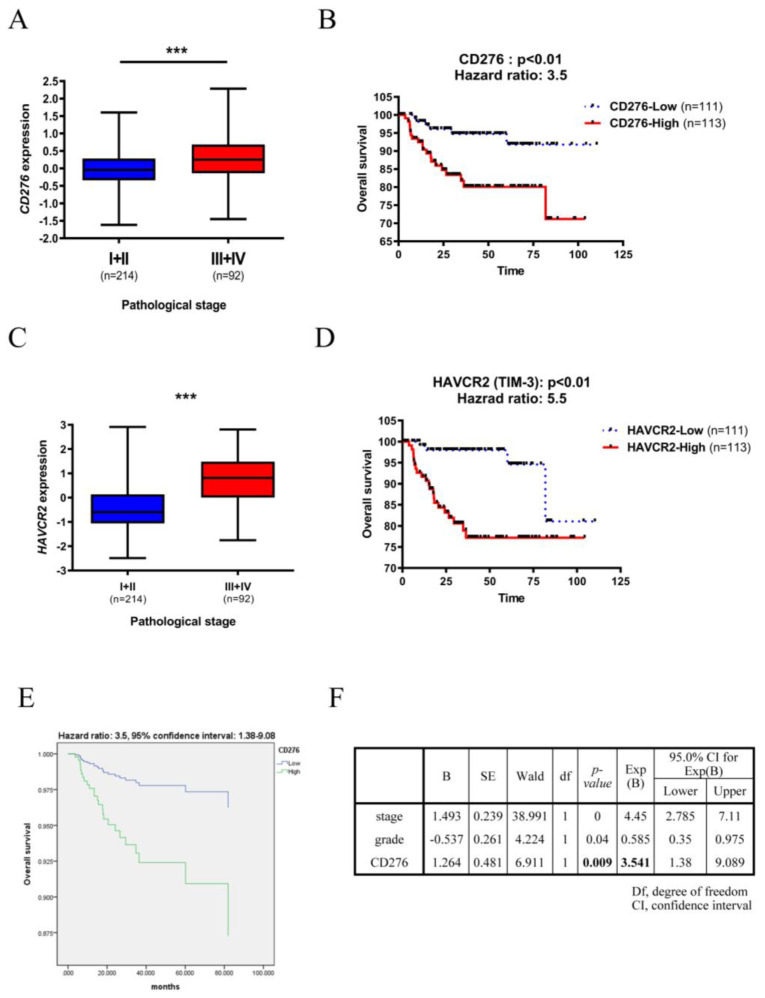
*CD276* and *TIM-3* expression positively correlates with the poor prognosis of UC. GEO dataset (GSE32894) was analyzed to determine the clinical significance of UC. (**A**) CD276 (**C**) *HAVCR2* (*TIM-3*) expression was grouped by early pathological stage (I + II) and late-stage (III + IV). (**B**,**D**) Overall survival of 224 UC patients was grouped by lower and higher (**B**) *CD276* (**D**) *TIM3* expression using the median value as a cut-off. Kaplan-Meier survival analysis was performed. (**E**) Multivariate Cox regression survival plot of 224 patients with UC was grouped by lower (*n* = 111) and higher (*n* = 113) *CD276* expression using the median value as the cut-off. (**F**) Results of the *CD276* multivariate analysis. *** *p* < 0.001.

To determine the role of typical immune checkpoints in the progression of UC, we examined whether *CD276, CD80, ICOS, CTLA4, PD-1* (*PDCD1*), *TIM-3* (*HAVCR2*), and *LAG3*, expression was correlated with pathological stage and overall survival of 308 patients with UC using a GEO dataset (GSE32894) [24]. Higher *CD276, CD80, ICOS, TIM-3*, and *LAG3* expression significantly correlated with the advanced pathological stage and poor UC prognosis (Figure 1A–D and Figure S1). Among these immune checkpoints, high *CD276* and *TIM-3* expression was correlated with high risk of UC mortality with 3.5 and 5.5 hazard ratio, respectively. Notably, *PD-L1* (*CD274*), *PD-1* and *CTLA4* did not significantly correlate with poor outcomes in patients with UC (Supplementary Figure S1A), indicating that typical immune checkpoints might not be the most crucial immunomodulatory molecules for UC immune tolerance. This result suggested that *PD-L1* may be a minor regulator of immune tolerance in UC. Since the expression level of these immune checkpoints was positively correlated with UC stage, we perform multivariate Cox regression survival analyses to explore the tumor stage and tumor grade independent prognosis markers. Although *TIM-3* has the highest hazard ratio (H.R. = 5.5) in univariate analysis, *TIM-3* was not significant correlated with overall survival in multivariate analysis (Supplementary Table S1). Interestingly, *CD276* is an independent prognostic factor for UC patient survival (Figure 1E,F).

**Figure 2:**
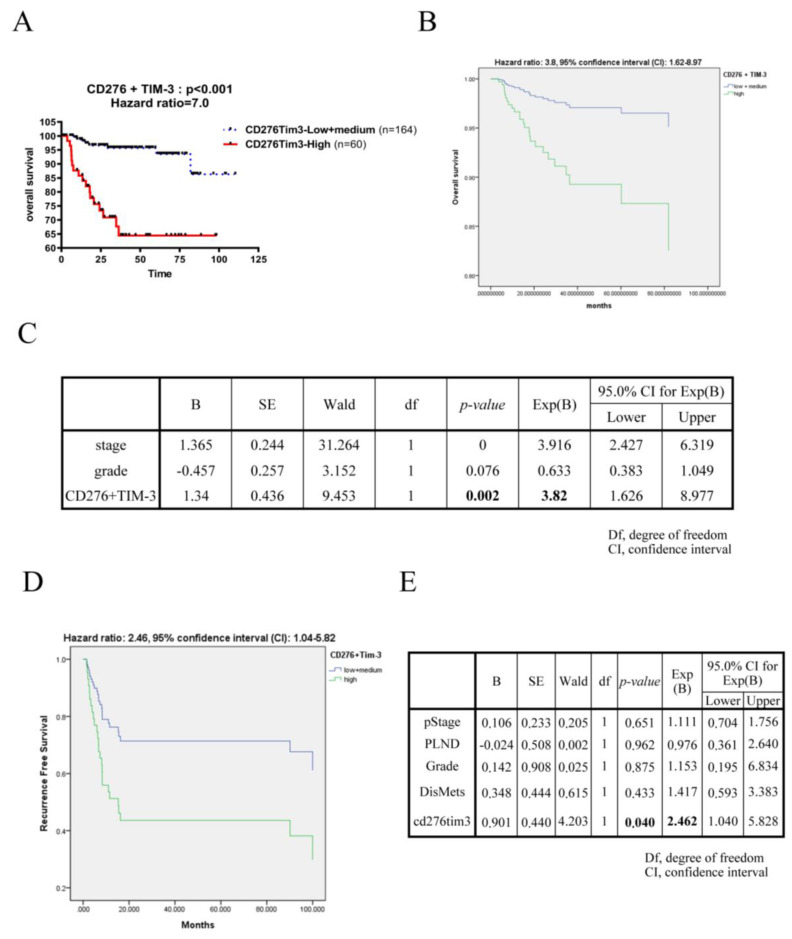
The combination of *CD276* and *TIM-3* was an independently poor prognostic biomarker of UC. (**A**) Univariate overall survival of 224 UC patients from GSE32894 was grouped by double-negative low, single-positive medium and double-positive high *CD276* and *TIM3* expression using the median value as the cut-off. The *CD276TIM3* double-positive group consisted of 60 patients (red line), the *CD276* and *TIM3* single or double-negative group consisted of 164 patients (blue line). Kaplan-Meier survival analysis was performed. (**B**) Multivariate Cox regression survival plot of 224 UC patients from GSE32894 was grouped as (A) described. (**C**) Results of the multivariate analysis from (**B**). (**D**) Multivariate Cox regression recurrent-free survival plot of 93 UC patients from GSE31684 was grouped by lower + medium (*n* = 63) and higher (*n* = 30) *CD276TIM3* as (**A**) described. (**E**) Results of the multivariate analysis from (**D**). The *p* values were calculated by the log-rank test.

In order to enhance the specificity and accuracy of UC prognostic factor, we combine *CD276* with different immune checkpoints which were significant in univariate analysis to perform survival analysis. In Figure 2A and Figure S2, the *CD276* and *TIM-3* double-positive group have the highest risk of UC mortality then double-negative group (hazard ratio = 7.0). By multivariate Cox regression survival analyses, *CD276* and *TIM-3* double-positive group shows poor prognosis regardless of tumor stage and tumor grade (Figure 2B,C and Supplementary Table S2). These data reveal that UC patients with simultaneously high *CD276* and *TIM-3* expression in tumor have significantly reduced overall survival at the highest risk (hazard ratio = 3.8), which was verified by regression model in dataset GSE13507 and GSE31684, showing 0.69 and 0.97 accuracy rates, respectively, to predict the mortality of these datasets. Furthermore, the double-positive combinatorial biomarker of *CD276-TIM3* shows an independently prognostic role for UC recurrence in GSE31684 dataset (Figure 2D,E). These data show that the combination of *CD276* and *TIM-3* is an independently prognostic biomarker for UC survival and recurrence and may be a valuable target for UC progression.

**Figure 3:**
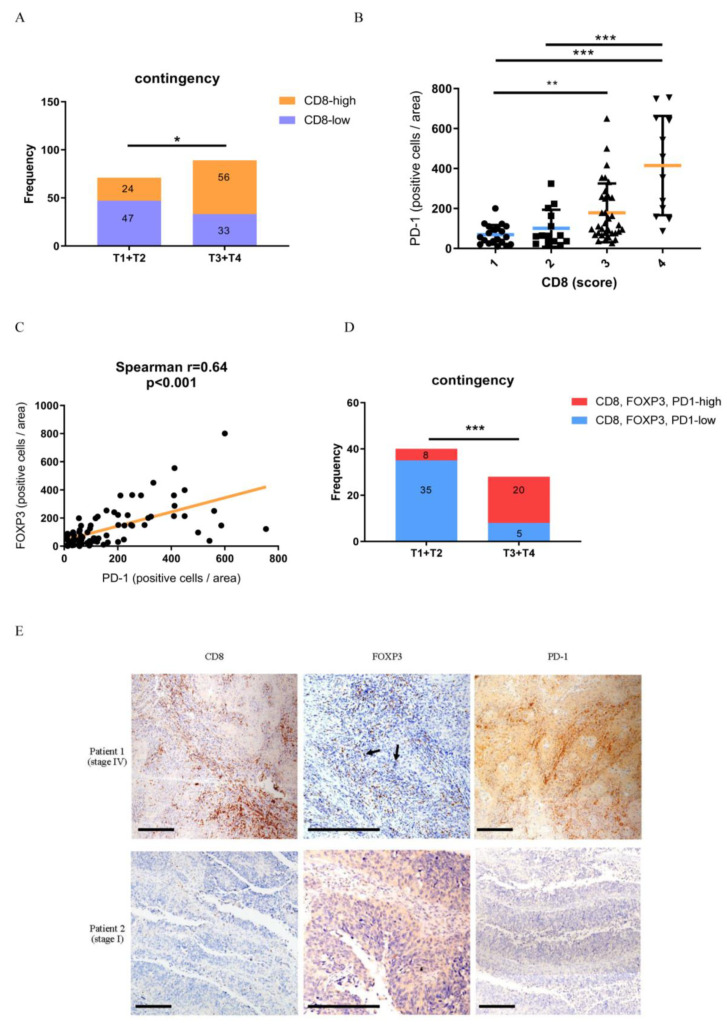
A subpopulation of patients with late-stage UC co-express CD8, PD-1, and FOXP3 in the tumor microenvironment. (**A**) IHC score of CD8 from 80 UC tumor specimens was divided into two groups by early (I + II) and late (III + IV) stage. The median value was used as a cut-off for CD8 expression. Analysis was performed using the Chi-square test. *, *p* < 0.05. (**B**) IHC score of CD8 and PD-1 from 37 late-stage UC tumor specimens. The PD-1 expression level was analyzed by one-way ANOVA. *, *p* < 0.05; **, *p* < 0.01. (**C**) The Spearman correlation test was used to analyze IHC scores of PD-1 and FOXP3. (**D**) IHC scores of CD8, FOXP3, and PD-1 from 40 UC tumor specimens were divided into two groups by early (I + II) and late (III + IV) stage. The median value was used as a cut-off for expression. Analysis was performed using the Chi-square test. ***, *p* < 0.001. (**E**) Representative images of CD8, FOXP3, and PD-1 IHC in patients with late-stage and early-stage UC. The positive signal of TILs is presented as brown “dots” indicated by arrow in the tumor section. Scale bar: 200 μM. UC, urothelial carcinoma; PD-1, programmed cell death protein 1; IHC, immunohistochemistry; TILs, tumor-infiltrating lymphocytes.

In recent years, TILs have been used to evaluate the prognosis of several tumors. However, some controversial issues need to be addressed, and the evaluation of TILs remains undefined. Recent studies have proposed that tissue-resident memory CD8+ T cells (Trm) share similar characteristics and gene profiles to TILs [29,30]. In contrast, Tregs play a pivotal role in immune tolerance and T-cell exhaustion. We analyzed 80 specimens of typical primary UC by performing IHC for T-cell subsets with antibodies against CD8 and Foxp3. Interestingly, a higher number of CD8+ TILs were present in late-stage UC (T3 + T4) than in early-stage UC (T1 + T2) (Figure 3A). Infiltration by these T-cell types in each compartment was quantitated numerically by blinded analysis of 5 hpf (0.0028 mm ^2^/hpf) and displayed as the average number of stained cells per hpf. Interestingly, we identified a subset of tumors (approximately 30%) that exhibited both large infiltrates of CD8+ T cells and highly expressed PD-1 during progression to the late-stage (Figure 3B). Among this tumor subset, the expression levels of PD-1 and FOXP3 showed a moderate positive correlation (Figure 3C). Finally, we notice that patients with simultaneously high CD8+, PD-1+ and FOXP3+ TILs infiltration significantly progressed to advanced UC when compared with early-stage UC (Figure 3D,E). These data indicate that a subpopulation of patients with late-stage UC overexpressing high levels of PD-1 did not naturally eliminate tumors despite an aggressive CTL microenvironment.

## 4. Discussion

The immune microenvironment plays a controversial role in tumor progression, and inhibition depends on the predominant immune cell subtype. Typically, tumors with strong Th1 and CTL components are easily eliminated by the immune system, resulting in a good prognosis. More importantly, we addressed a puzzling question in UC: Why are human tumors, which appear strongly immunogenic, not rejected by the host? The numbers of infiltrating lymphocytes and CTLs in some tumors are extremely high, and in some cases, exceed the number of tumor cells. Herein, we aimed to answer this question by analyzing the expression of immune checkpoints in UC specimens at different stages.

CD276 is a strong immunosuppressive checkpoint that is highly expressed in numerous solid tumors, which promotes the immune escape of tumor cells from the cytotoxic effects of interferon-gamma (IFN-γ) and tumor necrosis factor-alpha (TNF-α) [31]. Normal tissues reportedly exhibit low mRNA expression levels of *CD276*. However, relatively high *CD276* expression has been detected in various tumor tissues at all stages, including breast, cervical, colorectal, and prostate cancer [32,33,34]. Several research groups have proposed *CD276* as a UC tumor marker compared with normal tissue [35,36]. However, the prognostic role of *CD276* in UC progression remain unclear. In the present study, we observed that *CD276* mRNA was highly expressed in late-stage UC when compared with early-stage UC. Patients with high *CD276* expression have a poor UC prognosis independently.

TIM-3 belongs to the *TIM* gene family which binds to its ligand to inhibit cancer immunity by negatively modulating T-cell immunity [37]. Previous studies have proposed that TIM-3 was highly expressed in tumor and associated with tumor progression in colorectal and breast cancer [38,39]. Our results revealed that the mRNA expression level of TIM-3 was positive correlated advanced stage and overall survival in UC. Since most biomarker studies have been limited to a single biomarker [40], we are interested in utilizing combinatorial biomarkers to predict UC progression. Attractively, *CD276* and *TIM-3* in combination is an effective prognostic biomarker panel with highest hazard ratio value for univariate overall survival analysis (hazard ratio = 7.0). Multivariable Cox regression survival analysis also reveals *CD276-TIM3* in combination were independently associated with UC patient survival. To our knowledge, this study has shown for the first time that *CD276-TIM3* combination biomarkers act as independent prognostic biomarkers to predict UC outcome regardless of tumor stage and tumor grade.

UC patients have developed a local recurrence rate of about 22–47% [41]. Several risk factors have been identified to predict UC recurrence, including the number of tumors, tumor location, tumor stage, etc. [42]. By Cox regression analysis (multivariate), we evaluate the prognostic potential of *CD276-TIM3* combinatorial biomarker for UC recurrence in relation to pathological stage, lymph node metastasis, tumor grade, and distant metastasis. Our data revealed that only *CD276-TIM3* combinatorial biomarker displayed an independent prognostic value for UC recurrence. Collectively, these finding support the utility of *CD276-TIM3* combinatorial biomarker as a useful prognosis predictor for UC survival and recurrence.

Considering that the activity of immune checkpoints is associated with CD8+ T cell density [43], we explore the relationship of Treg and CTLs in different stage of UC specimens. Using IHC staining, we explored the relationship between immune checkpoint protein expression and UC progression. Given the high rate of CD8+, FOXP3+, and PD-1+ TILs in advanced-stage specimens, our data clarified why late-stage UC was not eliminated despite the high infiltration of CD8+ T cells in the microenvironment. Herein, approximately 30% of late-stage UC tumor specimens exhibited high CD8, PD-1, and FOXP3 expression levels, which could be improved by anti-PD-1 or anti-PDL1 therapy.

Intravesical instillation of bacillus Calmette–Guérin (BCG) and anti-PD-1/PD-L1 immune checkpoint blockade have been successfully employed for UC therapy. However, previous studies have proposed that UC may cause certain immune defects, especially modulating lymphocytes [44]. Approximately 30% of patients showed a negative response to BCG, and up to 74% of initial responders experienced recurrence [45], which could be related to immune tolerance in tumor microenvironment. Tumors with low T-cell infiltration, such as breast cancer, have been immunologically defined as “cold tumors” and are reportedly less responsive to single-agent checkpoint treatment [46]. A cold tumor can be converted to a “hot tumor” by cisplatin-based chemotherapy, radiotherapy, DC activators (such as agonist CD40 antibodies), and T-cell agonists (such as OX40 or CD137 antibodies) [47,48]. Our data indicate that fewer TILs were present in the tumor microenvironment during early-stage UC than in late-stage UC. Therefore, standard treaetments for early-stage UC, potentially converting cold tumors into hot tumors, followed by immune checkpoint therapy may benefit our patients.

In summary, our findings provide a better prognosis prediction by combining *CD276-TIM3* immune checkpoint markers in UC. Higher rates of CD8+, FOXP3+, and PD-1+ TILs in advanced-stage specimens and fewer TILs in the tumor microenvironment of early-stage UC were noticed. The tumor microenvironments provide a rationale for a combination therapy with standard drugs for early-stage UC, to potentially convert cold tumors into hot tumors, followed by immune checkpoint therapy.

## Data Availability

Publicly available datasets were analyzed in this study. This data can be found here: [https://www.ncbi.nlm.nih.gov/geo/query/acc.cgi?acc=GSE32894/GSE32894] assessed on 20 October 2021, [https://www.ncbi.nlm.nih.gov/geo/query/acc.cgi?acc=GSE13507/GSE13507] assessed on 20 October 2021, and [https://www.ncbi.nlm.nih.gov/geo/query/acc.cgi?acc=GSE31684/GSE31684] assessed on 20 October 2021.

## Supplementary Materials

The following are available online at https://www.mdpi.com/article/10.3390/biomedicines10010008/s1, Figure S1: Prognostic analysis of immune checkpoints., Table S1: Multivariate Cox regression survival plot of 224 UC patients from GSE32894 dataset., Figure S2: Kaplan-Meier survival analysis of combinatorial immune checkpoints. Table S2: Cox-regression survival analysis of combinatorial immune checkpoints.
