# Supplementary material for: Combination Biomarker of Immune Checkpoints Predict Prognosis of Urothelial Carcinoma
Source: Biomedicines. 2021 Dec 22;10(1):8. doi: 10.3390/biomedicines10010008 (PMC8772792; doi:10.3390/biomedicines10010008)
Supplement: Supplementary file 1 [file biomedicines-10-00008-s001.zip › biomedicines-1450441-supplementary.pdf]

Supplementary Figure S1

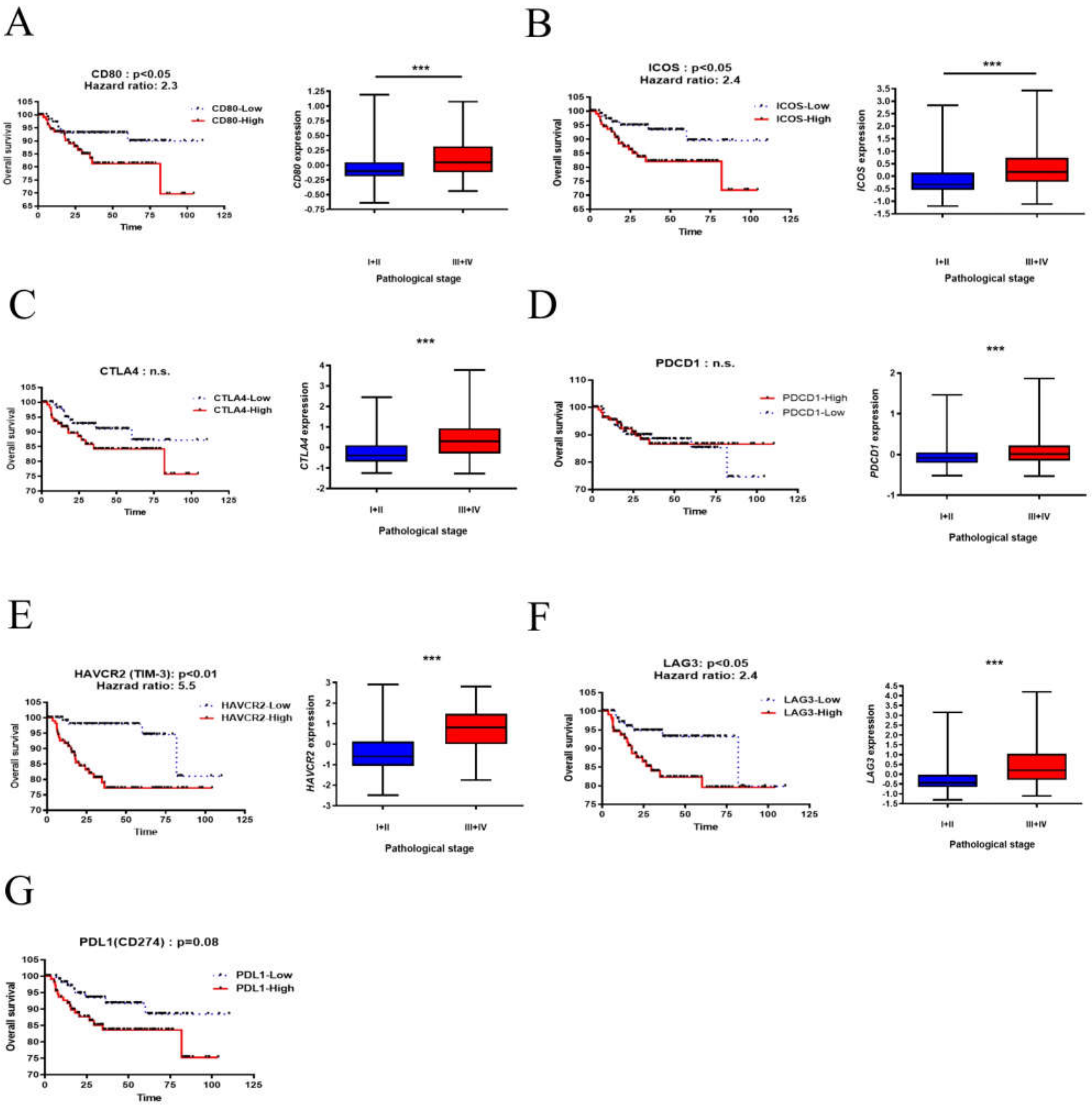

Figure S1. Prognostic analysis of immune checkpoints. 224 UC patients from GSE32894 dataset was grouped by lower and higher (**A**) *CD80* (**B**) *ICOS* (**C**) *CTLA4* (**D**) *PDCD1* (PD-1) (**E**) *HAVCR2* (TIM3) (**F**) *LAG3* and (**G**) *CD276* (PDL1) expression using the median value as a cut-off. Left panel: Kaplan-Meier survival analysis was performed. Right panel: The expression value of immunomodulatory molecules were grouped by early pathological stage (I+II, n=214) and late-stage (III+IV, n=92).*** p<0.001.

Table S1. Multivariate Cox regression survival plot of 224 UC patients from GSE32894 dataset was grouped by lower and higher expression of immune checkpoints, including *CD276*, *CD80*, *ICOS*, *CTLA4*, *PDCD1* (PD-1), *HAVCR2* (TIM3) and

	B	SE	Wald	df	*p-value*	Exp(B)	95.0% CI for Exp(B)	
							Lower	Upper
stage	1.493	.239	38.991	1	.000	4.450	2.785	7.110
grade	-.537	.261	4.224	1	.040	.585	.350	.975
CD276	1.264	.481	6.911	1	**.009**	**3.541**	1.380	9.089
stage	1.429	.222	41.269	1	.000	4.175	2.699	6.456
grade	-.391	.256	2.331	1	.127	.676	.409	1.117
CD80	.398	.436	.834	1	.361	1.489	.634	3.499
stage	1.431	.222	41.475	1	.000	4.184	2.707	6.468
grade	-.364	.258	1.986	1	.159	.695	.419	1.153
ICOS	.396	.464	.729	1	.393	1.486	.598	3.692
stage	1.445	.225	41.428	1	.000	4.242	2.732	6.586
grade	-.411	.255	2.591	1	.107	.663	.402	1.094
CTLA4	.275	.435	.399	1	.528	1.316	.561	3.085
stage	1.406	.212	43.979	1	.000	4.080	2.693	6.182
grade	-.420	.255	2.709	1	.100	.657	.398	1.084
PDCD1	-.136	.413	.108	1	.742	.873	.389	1.960
stage	1.402	.236	35.400	1	.000	4.063	2.560	6.448
grade	-.313	.256	1.488	1	.223	.731	.443	1.209
HAVCR2	.900	.556	2.617	1	.106	2.460	.827	7.318
stage	1.440	.224	41.428	1	.000	4.222	2.723	6.546
grade	-.362	.255	2.012	1	.156	.696	.422	1.148
LAG3	.490	.462	1.123	1	.289	1.632	.660	4.035

Df, degree of freedom
CI, confidence interval

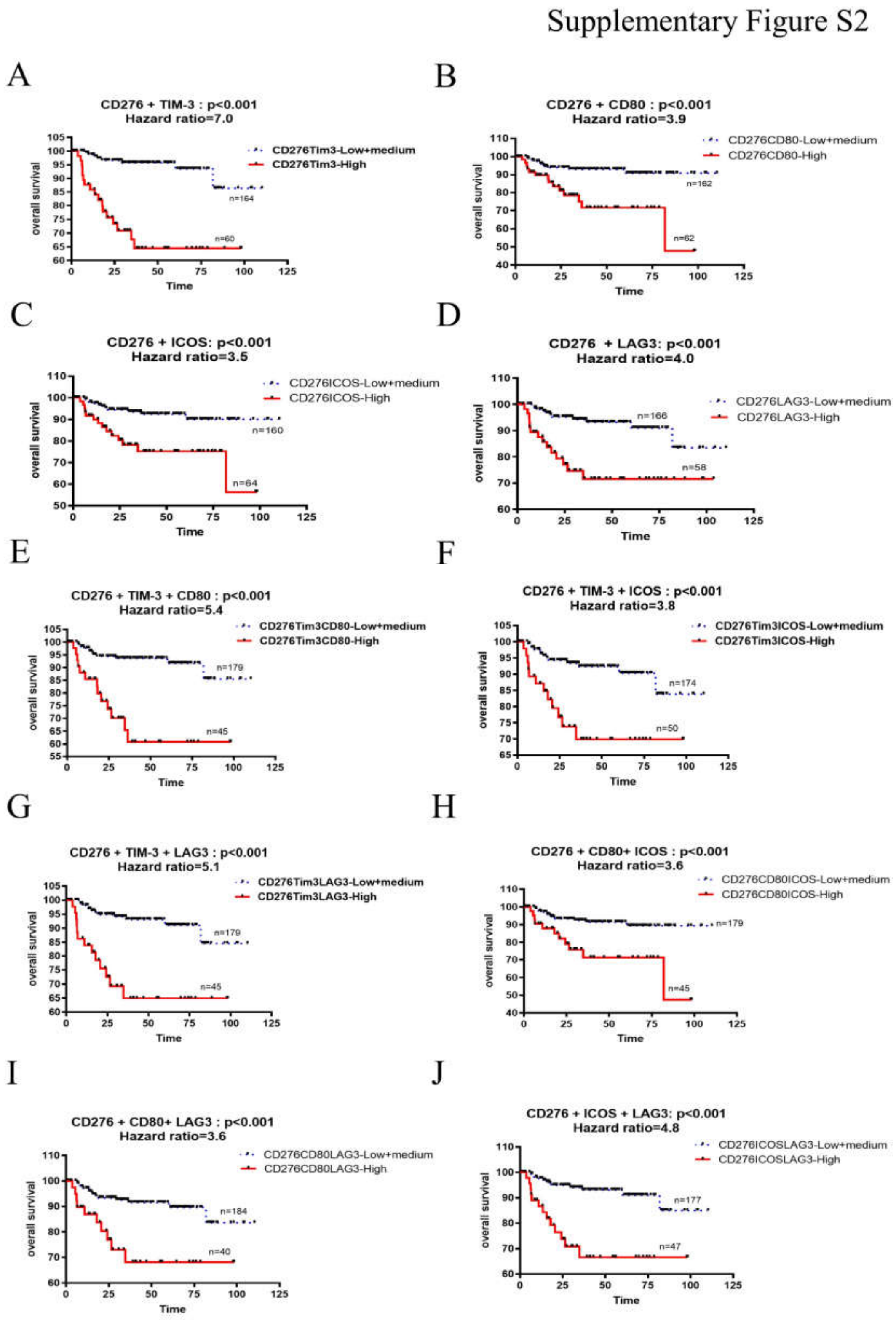

Figure S2. Kaplan-Meier survival analysis of combinatorial immune checkpoints. Univariate overall survival of 224 UC patients from GSE32894 was grouped by double/triple-negative as low expression, single-positive as medium expression and double/triple-positive as high expression using the median value as the cut-off.

Table S2. Cox-regression survival analysis of combinatorial immune checkpoints. Multivariate overall survival of 224 UC patients from GSE32894 was grouped by double/triple-negative as low expression, single-positive as medium expression and double/triple-positive as high expression using the median value as the cut-off.

	B	SE	Wald	df	*p-value*	Exp(B)	95.0% CI for Exp(B)	
							Lower	Upper
stage	1.365	.244	31.264	1	.000	3.916	2.427	6.319
grade	-.457	.257	3.152	1	.076	.633	.383	1.049
cd276tim3	1.340	.436	9.453	1	**.002**	**3.820**	1.626	8.977
stage	1.398	.229	37.227	1	.000	4.048	2.583	6.343
grade	-.441	.256	2.965	1	.085	.643	.390	1.063
cd276cd80	.991	.407	5.918	1	.015	2.694	1.212	5.988
stage	1.462	.234	39.018	1	.000	4.316	2.728	6.830
grade	-.442	.255	3.003	1	.083	.642	.390	1.060
cd276icos	.992	.407	5.938	1	.015	2.698	1.214	5.993
stage	1.441	.230	39.322	1	.000	4.226	2.693	6.630
grade	-.427	.256	2.778	1	.096	.653	.395	1.078
cd276lag3	.927	.403	5.296	1	.021	2.527	1.147	5.565
stage	1.360	.233	34.185	1	.000	3.897	2.470	6.149
grade	-.432	.254	2.879	1	.090	.649	.394	1.069
cd276tim3cd80	.925	.408	5.126	1	.024	2.521	1.132	5.613
stage	1.418	.228	38.625	1	.000	4.127	2.639	6.454
grade	-.414	.256	2.623	1	.105	.661	.400	1.091
cd276tim3icos	.822	.402	4.181	1	.041	2.276	1.035	5.004
stage	1.403	.233	36.161	1	.000	4.068	2.575	6.427
grade	-.414	.254	2.654	1	.103	.661	.402	1.088
cd276tim3lag3	1.081	.403	7.201	1	.007	2.947	1.338	6.490
stage	1.410	.226	38.990	1	.000	4.096	2.631	6.377
grade	-.425	.255	2.768	1	.096	.654	.397	1.079
cd276cd80icos	.819	.405	4.091	1	.043	2.268	1.026	5.016
stage	1.407	.223	39.621	1	.000	4.083	2.635	6.327
grade	-.417	.255	2.671	1	.102	.659	.399	1.087
cd276cd80lag3	.666	.410	2.632	1	.105	1.946	.871	4.350
stage	1.416	.233	36.875	1	.000	4.120	2.609	6.507
grade	-.436	.252	2.990	1	.084	.647	.395	1.060
cd276icoslag3	1.048	.403	6.753	1	.009	2.852	1.294	6.287

Df, degree of freedom
CI, confidence interval
